# Patients’ Perceptions of Portal Use Across Care Settings: Qualitative Study

**DOI:** 10.2196/13126

**Published:** 2019-06-06

**Authors:** Ann Scheck McAlearney, Cynthia J Sieck, Alice Gaughan, Naleef Fareed, Jaclyn Volney, Timothy R Huerta

**Affiliations:** 1 Department of Family Medicine College of Medicine The Ohio State University Columbus, OH United States; 2 CATALYST (Center for the Advancement of Team Science, Analytics, and Systems Thinking) College of Medicine The Ohio State University Columbus, OH United States; 3 Department of Biomedical Informatics College of Medicine The Ohio State University Columbus, OH United States; 4 Division of Health Services Management and Policy College of Public Health The Ohio State University Columbus, OH United States

**Keywords:** patient portals, hospitalization, medical informatics, implementation, engagement, ambulatory care

## Abstract

**Background:**

Patient portals are a promising instrument to improve patient-centered care, as they provide patients information and tools that can help them better manage their health. The implementation of portals in both the inpatient and outpatient setting gives health care providers an opportunity to support patients both during hospitalization and after discharge. Thus, there is a need to better understand how inpatient and outpatient portals are used across care contexts.

**Objective:**

This study aimed to examine patients’ perceptions of using inpatient and outpatient portals across the care settings, including how they used the portals and the benefits and concerns associated with portal use.

**Methods:**

This study was conducted in a large Midwestern academic medical center consisting of seven hospitals. We interviewed 120 patients who had used an inpatient portal during their hospitalization, at 15 days and 6 months postdischarge, to determine their perspectives of portal use in both hospital and outpatient settings. Interview transcripts were analyzed inductively and deductively by using team coding processes consistent with a grounded theory approach.

**Results:**

Interviews focused on three main areas of portal use: experience with the portal features, perceived benefits, and concerns. Responses at 15 days (n=60) and 6 months (n=60) postdischarge were consistent with respect to perceptions about portal use. Patients identified viewing their health information, managing their schedule, and communicating with providers as notable activities. Convenience, access to information, and better engagement in care were indicated as benefits. Concerns were related to technology issues and privacy/security risks.

**Conclusions:**

Implementation of inpatient portals as a complement to outpatient portals is increasing and can enable patients to better manage aspects of their care. Although care processes vary substantively across settings, the benefits of convenience, improved access to information, and better engagement in care provide opportunities for portal use across care settings to support patient-centered care.

## Introduction

The focus on patient-centered health care delivery has made patient portals a promising medium through which patients can gain access to their health information [1,2]. Patients can use the tools and information portals provide to better manage their health, potentially increasing engagement with providers and improving the process of care delivery [3-5].

Inpatient and outpatient portals are usually offered by a health care provider or institution and linked to an electronic health record system. Outpatient portals, typically Web-based, offer patients information focused on outpatient care including access to a health summary, medication listing, immunizations, patient health data entry, appointment tracking, secure messaging, and patient financial account management information. Portals are now increasingly offered in the inpatient setting to hospitalized patients, providing customized information for a patient’s inpatient stay including an expected daily care plan, secure messaging with the care team, a notes feature, and educational materials.

Although inpatient and outpatient portals are independent applications, patient information is available through a common electronic health record platform. Together, these two types of portals can provide complete access to health information and providers across care settings. For example, a hospitalized patient could use educational materials, track test results, and make notes during his/her inpatient stay. After discharge, an outpatient portal provides information about the patient’s recent hospital stay [6] and can be used to facilitate ongoing management of laboratory and test results as well as continued communication with the patient’s care team.

Studies examining portal use primarily focus on a single application: inpatient or outpatient. The evidence on patient use of inpatient and outpatient portals converges on the benefits of portal use, including improved patient-provider interactions, higher awareness of care, and better adherence to care regimens; it also comparably notes portal shortcomings including fears of privacy loss, concerns about results comprehension, and technical challenges with portal use [7]. A recent systematic review of portal use, however, highlighted significantly more evidence regarding outpatient portal use compared with inpatient portals, suggesting the need for more empirical research on the newer inpatient portal technology [8]. Furthermore, there is currently a gap in research that examines the use of patient portals across care settings [9,10]. As such, there remains a clear need for more research to understand both how portal use is evolving in the health care system and how patients perceive portal use across care settings.

This study was conducted in response to the need to understand how patient portals function in different contexts. Our study aims to describe patients’ experiences with both inpatient and outpatient portals, including exploring how patients’ perceptions compare across settings. This study was approved by The Ohio State University’s Institutional Review Board.

## Methods

### Study Setting

Our study was conducted in a large Midwestern academic medical center consisting of seven hospitals. The center began offering the MyChart outpatient portal in 2011 (Epic Systems, Verona, WI). Patients can access MyChart via the website on a desktop computer or a mobile app on electronic devices (eg, smartphones). In 2013, the academic medical center introduced MyChart Bedside (MCB), a companion portal tailored to the inpatient environment, in a few pilot units before introducing MCB system-wide in 2016. Patients can access MCB on academic medical center–provided tablets during their hospital stay and are able to access their inpatient data via the outpatient portal after discharge.

As shown in Table 1, both MyChart and MCB offer features related to scheduling, personal health information, and health education. In addition, both portals offer slightly different ways to communicate with health care providers. MyChart, the outpatient portal, allows a patient to selectively communicate with an individual provider, either their primary care provider or a specialist; in contrast, messages sent via MCB, the inpatient portal, are sent to the entire care team assigned to that patient. Additional features that are specific to each portal are compared in Table 1.

**Table 1:** Comparison of features of inpatient and outpatient portals.

Portal feature	Feature description	Inpatient portal function (MyChart Bedside)	Outpatient portal function (MyChart)
Scheduling	View schedule for the day (inpatient) or schedule appointments (outpatient)	Happening Soon	Visits
Health information	View personal health information including biometric data and medications	My Health	My Medical Record
Health education	Read health education materials assigned by health care providers	To Learn	Resources
Secure messaging	Communicate with care team (inpatient) or individual providers (outpatient)	Messages	Messaging
Refill medications	Request refill of prescribed medications	N/A^a^	Request a Refill
Pay bill	Pay inpatient or outpatient bill	N/A	Billing
Set communication preferences	Select frequency of email or text communication	N/A	Preferences
View care team	View names and pictures of all members of inpatient care team assigned to the patient at each shift	Taking Care of Me	N/A
Order meals	Select menu items from the prescribed diet	Dining on Demand	N/A
Request a service or item	Request services such as pastoral care or delivery of a newspaper	I Would Like	N/A
Make notes	Type items the patient wishes to remember	Note to Self	N/A

^a^N/A: not applicable.

**Table 2:** Participant demographics.

Demographic	Phase 1 (15 days postdischarge)	Phase 2 (6 months postdischarge)
Total participants, n	60	60
Age (years), mean (SD)	49.4 (14.3)	49.4 (13.7)
Male participants, n (%)	18 (30)	15 (25)

### Data Collection

We conducted two phases of 15-minute telephone interviews between January 2017 and May 2018, with a total of 120 patients who had been hospitalized at the academic medical center and used the inpatient portal during their recent stay. Phase 1 interviews were completed 15 days postdischarge, and Phase 2 interviews were completed 6 months postdischarge. We chose the timing of these interviews to capture immediate impressions in Phase 1 and longer-term impressions in Phase 2. Furthermore, the timing of these interviews provided a window during which perspectives about both inpatient portal use (during hospitalization) and outpatient portal use (postdischarge) could be evaluated. Interview phases involved samples of different patients (Table 2), randomly selected from among all discharged patients who had consented to study participation. Patients were recruited for interviews based on their discharge dates within the appropriate time windows. We did not select our sample based on the level of portal use, as we wanted to understand the perspectives of all types of portal users. In addition, we intended to examine a range of patients’ perceptions about the portals, not limited by demographic characteristics or conditions.

Study investigators conducted interviews using semistructured guides designed to explore patient experiences with both the inpatient and outpatient portals. Patient interview guides are provided in Multimedia Appendix 1. These guides were developed by the research team and pilot tested on a sample of patient volunteers. The 15-day guide focused on the use of and experience with the inpatient portal during the patient’s recent hospital stay; the 6-month guide examined how the inpatient portal experience influenced outpatient portal use and asked about long-term use of the outpatient portal. All interviews were audio-recorded, transcribed verbatim, and de-identified for analysis.

### Data Analysis

Interview transcripts were analyzed both inductively and deductively, in accordance with rigorous qualitative methods [11,12]. First, a preliminary coding dictionary was developed based on questions asked in the interview guide. Next, a sample of four interview transcripts were coded by four members of the research team (AM, CS, AG, and TW) to refine the coding dictionary and explore the emergence of new codes in the data. Using the refined coding dictionary, all interview transcripts were coded by two members of the research team who held frequent meetings to ensure consistency of coding and agreement about the definitions of new codes as they emerged during the coding process, consistent with our grounded theory approach [13]. Development and refinement of the coding dictionary and all aspects of the coding and analysis were led by an experienced qualitative researcher (AM). ATLAS.ti (version 6.0; ATLAS.ti Scientific Software Development GmbH, Berlin, Germany) qualitative data analysis software was used to support this analysis.

## Results

### Overview

Three main topics were discussed by patients when they were asked about their experience with patient portals: experience with the portals, perceived benefits of portal use, and concerns about portals. Patients’ responses to interview questions at 15 days and 6 months postdischarge did not vary appreciably from each other; as a result, the findings we present do not distinguish between interview time frames.

### Patients’ Experiences with Portal Use

When describing their use of portals across care settings, patients primarily discussed three main activities: viewing health information, managing schedules, and communicating with providers. Patients’ uses of these features are discussed below with additional exemplary quotes presented in Textbox 1.

**Patients’ experiences with portal use (portal features used and examples of use).**

**Viewing health information**

Examples of inpatient portal use:

- I just used it to look at what my medicines were and what kind of, and then what the medicines were all about, what was in them and everything like that. [Patient #610]
- I got to look at my charts and see my bloodwork and see how things were going. [Patient #627]

Examples of outpatient portal use:

- To find out my medical condition, the symptoms…any details about a medical condition, you can pretty much trace it back and go through that system, and find out things. [Patient #613]
- I can now see test results and also just whatever medical information that’s helpful. [Patient #610]

**Managing schedules**

Examples of inpatient portal use:

- I could kind of know what the plan was gonna be the days that I was in the hospital. [Patient #623]
- The ability to stay on track with what your schedule is and know what your treatment plan is. [Patient #620]

Examples of outpatient portal use:

- I’ll look up my appointments sometimes if I have a scheduling conflict then I utilize that tab for a more direct access to my providers. [Patient #619]
- I use it to make appointments and change appointments...[Patient #624]

**Communicating with providers**

Examples of inpatient portal use:

- What I liked about it was that I was able to correspond with the doctors, nurses. [Patient #614]
- I messaged my doctors to see what the…cause like when you look at your bloodwork it’s got this scale and it’s got numbers and I didn’t know what they were. So I, it let me hit a button and ask them questions: “What does this mean?” and “Am I okay? Am I going to live?” [Patient #627]

Examples of outpatient portal use:

- I can e-mail the doctors and they, you know, e-mail me back. I think it’s a very good tool. [Patient #608]
- Well, it’s communication with my doctor, my regular doctor. He’ll put notes in there and I can put notes back to him. [Patient #626]

#### Viewing Health Information

Views of laboratory and test results as well as features to learn more about what these results meant were important portal features used by patients. In the inpatient setting, patients could view results as soon as they became available and track multiple tests throughout the day. For example, one patient reflected,

I used it daily, probably a few times a day. When I have a new test, I just check when it’s there, or if the results are back or not.

Patient #104

In the outpatient environment, patients could also view results as soon as they were available, but their portal use also allowed them to monitor changes over time:

I use it to look up new lab results when they’re available, and also sometimes to look up older ones to kind of look at the trends.

Patient #624

#### Managing Schedules

Both in the hospital and outpatient settings, patients reported using the schedule feature. Inpatients appreciated knowing when tests and medication administrations were scheduled:

I liked the schedule that was on there of what the various times of when I was going to receive my medicine and everything. That way I knew what was coming.

Patient #610

In the outpatient setting, patients noted frequently using this scheduling feature, “to keep track of appointments” (Patient #228).

#### Communicating With Providers

The ability to communicate with providers was a feature used in both portals. One patient described their use of the secure messaging feature while hospitalized:

If you didn’t understand something from the doctor, you could message him and ask him.

Patient #625

In the inpatient environment, however, not all patients reported the need for another way to communicate with their providers, given the frequency of in-person interactions with their care team:

They [care team members] came in every day and told me what the test results were, so I didn’t spend a lot of time on that.

Patient #112

In contrast, in the outpatient environment, the majority of patients used the portal to communicate with providers:

Well, since I’ve gotten out of the hospital, just to stay in contact with my doctor, or doctor’s visits that I might not even have to see because I was able to communicate with him and find things out without going to the doctor’s office.

Patient #626

### Perceived Benefits of Portal Use

Patients in our study noted benefits from their use of portals in both the inpatient and outpatient settings, and these centered around three themes: convenience, improved access to information, and better engagement in care. Below, we describe the perceived benefits in these areas and present additional supporting quotations in Textbox 2.

**Perceived benefits of portal use (with examples of use).**

**Convenience**

Examples of inpatient portal use:

- I’d say it was nice because it made it easier for me to, pretty much just whenever I wanted to, or whenever it was convenient for me. [Patient #202]
- That you could see your lab results and medications and everything. I liked it. I mean it was easy to use. [Patient #625]

Examples of outpatient portal use:

- Well MyChart to me is efficient. It’s what I need to get through my day and I can do it at work, anywhere I’m at any time of day. So it’s extremely beneficial. [Patient #626]
- You do not have to be put on hold for MyChart. It’s all just right there and there is no other step or hoop to jump through to get the answers that you need for that. [Patient #607]

**Improved access to information**

Examples of inpatient portal use:

- The knowledge of what was actually going on and keeping up with my medications as they were changing and stuff. All that. That’s what I really liked about it was the ability to help me keep up. [Patient #617]
- One thing that I just remembered that I really liked was like it would tell me like when I might be getting discharged and how many days I’ve been in the hospital as well because sometimes you tend to lose track. [Patient #227]

Examples of outpatient portal use:

- I liked it so well is because my health was quickly changing and I really didn’t understand all of why it was changing so quickly. And it helped me to understand those changes. [Patient #617]
- I needed to remember appointments because I was having appointments here and there and all over the hospital and all over campus. I was all over campus and I needed to remember what time, what day where I was supposed to be, and then what the results were from each appointment that I had at that time. [Patient #618]

**Better engagement in care**

Examples of inpatient portal use:

- I was able to read, read my test results, usually before they [the care team] came in, and I was able to figure out questions I wanted to ask them before they got there. [Patient #109]
- I feel like I’m an active participant. [Patient #229]

Examples of outpatient portal use:

- Every time I open up my email, I always pay attention and make sure that I don’t miss the emails saying that yeah when you message or you have something coming from MyChart. And then I immediately go and log in and see what areas and what has been added like new test results and new appointments. So that is always done to keep up to date and it is also extremely helpful. [Patient #122]
- It’s more just being one-on-one with the doctors, you know. If I need a refill I can e-mail the doctor or nurse or whatever and get it done or seeing results from tests, seeing my appointments. It’s a big help. [Patient #608]

#### Convenience

Patients noted many ways in which the portals in different care settings were convenient tools they could use on their own time. In the hospital, patients appreciated the ability to order their own meals:

Ordering food from [Dining on Demand]…that was all extremely helpful because…you have time to look at everything that’s there, and it allows you by far a much more varied menu from meal to meal, especially if you stay more than just a couple of days.

Patient #122

Another feature patients found convenient was the ability to review their own health information without waiting for a provider:

I could just log into it and look and see the results versus having to wait for the doctor or the nurse to come in and tell me the results.

Patient #107

The convenience of the outpatient portal was similarly valued, especially in comparison to not having access to a portal:

I find it far more convenient. Like when I’m waiting for lab results, to know that when that result is available, I can readily see it on there. I don’t have to wait potentially days for the doctor’s office to have time to go through all their results and contact me, and then play phone tag with each other.

Patient #624

Another patient similarly explained,

I think having one central place to go to, to look up things like lab results and past appointments and surgery stuff is very helpful to have all in one place.

Patient #607

#### Improved Access to Information

Patients noted that portals provided improved access to information about their current health care plans and medical histories. For example, one patient described the benefits of having ready access to information while in the hospital:

Well it keeps you more in tune to your health. I mean it tells you, you got meds coming. It tells you why you’re taking the meds. There’s advice that’s good for everybody. Cause doctors fail to realize that we don’t understand all that medical terminology. Once they leave, I click on that little thing and it tells you what it’s for and…it tells you what it is and what the symptoms of it are and what they can do to fix it.

Patient #604

Similarly, in the outpatient setting, patients described using the outpatient portal to review discussions with their doctor:

The communication is very beneficial. I can also double check if I can’t remember what the doctor had explained with test results and things like that. I can always…look back at those.

Patient #621

#### Better Engagement in Care

Patients also reported that using the portals helped them fully engage with their care. For example, one patient told us that during their hospital stay, “It [MCB] gave me the information to ask the right questions” (Patient #209). Another patient specifically described the benefit of using the inpatient portal in contrast to not having it available:

You hit your call light. The nurse or the nurse assistant that comes in and they’re like, “What do you need?” But you don’t have a direct link to ask your doctor [like you do with the portal]…You have to wait 24 hours or so for the doctor to come down and see you. But that doctor is only going to be in there three or four minutes, not even four minutes, talking with you.

Patient #211

In the outpatient setting, patients similarly described how portals helped them engage in and take control of their health:

If it’s a person in charge of their own healthcare, and it’s not all responsibility of the medical facility anymore. But now it’s my responsibility because I have resources that I can go to that I can use.

Patient #613

Another patient explicitly noted the benefit of portal access across care settings:

I knew that once I was released from the hospital, I could go back, and if I wanted to, consult my doctors or send them a message about what my health was doing. So in that way it was nice.

Patient #156

### Concerns About Portal Use

There were two main categories of concerns reported by patient participants: technology-related issues and privacy and security issues.

#### Technology-Related Issues

Patients raised concerns about technology-related issues with both the MCB portal and the tablets. Several patients noted issues with the battery life of the tablets on which the portal was hosted. One patient reflected on the need for internet access outside the hospital to facilitate use of the outpatient version of the portal:

...if you’re a person that, you know, moves about a lot and maybe doesn’t have a…well you have to have internet for one thing. That’s one of the things, reasons why I didn’t actually use it is when I’m at my place, I don’t have internet. So unless I go to a library or I’m at my parents’, I don’t have any way of getting online.

Patient #602

Interestingly, patients occasionally suggested ways to improve the available features on the inpatient portal application. For example, one patient noted that the portal could be improved by providing patients the ability to send a secure message to a specified individual:

I wanted a specific doctor, or something to ask them a question, or a specific nurse or something. But it would always send it as a group message, and they said that they didn’t know who would respond to me.

Patient #130

#### Privacy and Security Issues

Patients also generally noted security and privacy issues related to portals:

Just like anything else, you know, you’re on the computer, people get in and get your business. So that’s the only thing I was worried about.

Patient #116

In the hospital setting, patients expressed concerns about using a hospital-provided tablet that would have to be returned and then used by another patient:

The one concern I had was that it was going to be wiped. So I took that…when I was finished with it, since it had my personal Kindle stuff on there. The one thing that could’ve been done, that sort of would’ve made me more confident, and I was confident enough to give it to them, was that actually had a button on the app…on the device itself, that I could erase it.

Patient #110

## Discussion

### Principal Findings

Portals have recently been incorporated across most aspects of patient care [14,15]. Although studies of outpatient portals have demonstrated that use can improve acceptance of preventive screening and adherence to disease management plans [16-19], the introduction of an inpatient portal can address the particular needs of a patient during hospitalization, such as the ability to communicate with the care team and know when to expect medications or treatments.

Patients in our study reported various ways in which they used portals across care settings as they became and remained involved in their care. For example, study participants noted ways in which increased access to information helped them better understand their care plan, such as through the use of scheduling features of both the inpatient and outpatient portals. While hospitalized, the inpatient portal informed patients when to expect medications or tests; once discharged, the outpatient portal allowed them to track appointments and medication refills. In both of these situations, participants noted that a better understanding of the flow of their care made them feel more engaged in the care process. The same appreciation for increased access to information through the portal was noted by patients who followed their laboratory results; patients reported valuing the ability to monitor results, as the results changed during their hospital stay, as well as the ability to track results over time in the outpatient portal. Together, this ecosystem of portals appears to help patients better understand their health trajectory, from moments of crisis (ie, hospitalization) to patterns of management (ie, outpatient care).

Our findings can be viewed using the Umar and Mundy model of patient empowerment that views health information technology as an underlying supportive mechanism for empowerment. Key elements of this model include patient access to information, knowledge development, patient participation as a partner, and functions that facilitate a sense of self-efficacy and align with our findings [20]. In our study, across settings, increased access to information appears to improve the experience of care as well as help patients to engage more fully in the care process, consistent with Umar and Mundy’s model that positioned patient portals as health information technology that supports empowerment. For instance, in the inpatient setting, lack of information has been identified as a concern that leads to increased anxiety for patients [8,21]. Inpatients in our study specifically noted the benefits of improved access to information, explaining that having access to the portal allowed them to keep track of the care process, thereby reflecting greater empowerment. In the outpatient setting, access to information is also critical for facilitating communication both in-person as well as between visits, with recent research showing that patient-provider in-person communication is enhanced when patients have viewed the results prior to their visit [22]. Communication with providers, which helps the patient become more of a partner in their care, is also an element identified in Umar and Mundy’s model that supports patient empowerment.

Our study is the first to explore patient perspectives of how inpatient and outpatient portals are used across care settings [2]. These findings provide preliminary evidence about the ways in which portal availability influences patients’ care experiences, noting convenience and access to information as benefits of both the inpatient and outpatient portals. As such, our results can serve as a foundation to an evidence-based approach that informs the development of portals that promote health, as recommended by Osborn and colleagues [23]. Further, patients described how portal use helped them stay engaged with and manage their health care, suggesting that portal access may have an important impact on factors such as patient self-efficacy [24,25], another important element of patient empowerment identified by Umar and Mundy [20], and the overall care experience [24,25] as patients attempt to better manage their health across care settings.

These study results are consistent with prior research that has highlighted barriers to portal use such as the need for technical support, training, and improved usability [23,26]; thus, addressing these barriers will be an important consideration as portal use extends across the health care continuum. Moreover, our study participants appreciated both the availability of the secure messaging feature and the speed with which providers could respond to their questions, consistent with the results of prior work that has found this feature useful [15,27,28]. At the same time, our study also highlights a continued need to address access to health information technology across care settings. Patients valued the portals, but some patients noted that, due to lack of internet access in the outpatient setting, their most consistent access to the portal was during hospitalization. Studies have found that approximately 70% of the US population has internet access [29], but such reports also note that access is strongly correlated with sociodemographic factors. Research has documented that disparities in internet access and use contribute to poorer health outcomes when comparing outcomes of individuals with consistent access [30-33], thus suggesting the importance of promoting health information technology access to improve health and health care.

Stakeholders in the health care system recognize the value of providing an integrated portal experience that spans care settings [14]. However, there are currently few theories or studies that effectively describe patients’ uses of technologies, and fewer still that can take into consideration their distinct needs across the health care continuum [26,34]. We found that having access to an inpatient portal integrated with an outpatient portal helped patients with the care process by providing different applications across care settings, from preventive care to diagnosis and treatment to disease management. Continuing to improve our understanding of the impact and influence of this new technology will be critical as we attempt to advance both the quality and patient-centeredness of care across care settings.

We noted several potential limitations to our study. First, our study examined patient perceptions related to the use of portals in a single health care system. Although the features available are common among most portals offered at other health care systems, policies regarding implementation and use may result in different experiences at different systems, and different portals in use may have features we could not study. Second, it is possible that patients’ recalls of their use of the portals were influenced by the time that had elapsed since their hospitalization. However, given the common ideas expressed at both 15 days and 6 months postdischarge, we believe the impact of time is likely minimal; instead, the stability of these findings across time may be a strength of our study. Third, patients who agreed to participate in our interviews might represent the most engaged group of patients. Although their use and perceptions may vary from those of less engaged patients, their insights are useful to inform future research and practical efforts to introduce and promote portal use. Finally, this qualitative study did not link patients’ comments to information about their hospital stay (eg, length of stay or location of treatment) or to their prior portal experience (eg, outpatient portal use prior to hospitalization). Further work that considers these dimensions of care and experience is needed to draw conclusions about the types of patients or clinical situations that can best benefit from different aspects of patient portal use.

Future studies of portal use should consider topics such as portal use patterns, including the relationships between concerns about portal use and subsequent use. There may also be idiosyncrasies of patient portals (eg, the timeliness of information availability in the outpatient portal after an inpatient discharge) that impact portal use across care settings, suggesting opportunities for continued study in this area. Further, examining the use of specific portal features and taking into account patient demographics as well as patient stage along the care continuum would be informative. As health care systems and researchers gain experience with portals that can span the continuum of care, it will be important to take advantage of opportunities to explore portal use in greater detail using both qualitative and quantitative methods.

### Conclusions

Development and implementation of inpatient portals as a companion to outpatient portals represent a nascent approach to portal use across the continuum of care. Although the processes of care are substantively different between inpatient and outpatient settings, among those who use inpatient portals, there is a clear preference for this technology. Common functions across the portal tools create familiarity and, by extension, increase engagement. Further, while these tools enable communication through messaging features, they are more useful because of the important information they provide patients to help them in their care journey. Convenience, improved access to information, and better engagement in care suggest specific aspects of portal use that can support patient engagement across care settings.

## Appendix

Multimedia Appendix 1 Semistructured patient interview guides.

## Abbreviations

MCB: MyChart Bedside
