# Supplementary material for: Patients’ Perceptions of Portal Use Across Care Settings: Qualitative Study
Source: J Med Internet Res. 2019 Jun 6;21(6):e13126. doi: 10.2196/13126 (PMC6592494; doi:10.2196/13126)
Supplement: Multimedia Appendix 1 [file jmir_v21i6e13126_app1.pdf]

Multimedia Appendix 1
Semi-structured patient interview guides

Patient interview guide – 15 days postdischarge

BACKGROUND
First, let us THANK YOU for agreeing to participate in our research project. I am (NAME) and I am a researcher from The Ohio State University. I am contacting you as part of a project we are doing to study MyChart Bedside (MCB), a personal health record offered by The Ohio State University Wexner Medical Center (OSUWMC) and the hospitals associated with the medical center.

As part of this study, we are interested in collecting information from patients about how they tracked their health conditions during their inpatient stay and later after they were discharged. Ultimately, this will help us in the future to understand the issues involved so that we can improve patient training and technology based on user experiences.

When you were recently in the hospital, you agreed to participate in this study, and I am calling you as a follow-up to see if you would be interested in participating in a brief telephone interview that will take about 20 minutes. In this interview, we will ask you several general and open-ended questions about your experience with managing your conditions when you were in the hospital, and your perceptions about what might be helpful. As a token of appreciation for your time participating in this interview, we will mail you a $15.00 gift card.

OVERVIEW OF INTERVIEW TOPICS
In this interview, I will ask you a series of open-ended questions to get your perspectives about different topics. As an overview, these topics are:

- Section 1: Patient Information
- Section 2: Managing Your Health
- Section 3: Experience with MyChart Bedside
- Section 4: Communication with Hospital Staff
- Section 5: Experience with MyChart Bedside Training

INTRODUCTION TO INTERVIEW
Before we begin the interview, I need to take you through an informed consent process. In particular, let me make sure that you understand that:

a. Your participation is completely voluntary. If you do choose to talk with us, you may decide to hang up at any time during this interview.
b. We consider this discussion to be confidential. Your participation is confidential in the sense that your name will not be used in any reports or articles.
c. We would also like to record the interview for the purposes of data collection for our research. The recording will not be used to identify you in any way.
d. Do you have any questions about our study or this interview process? Are you okay being recorded?

I will collect your address at the end of this call to mail you your gift card.

PATIENT (OR CAREGIVER) INTERVIEW QUESTIONS

Section 1: PATIENT INFORMATION

You may remember someone coming into your room with a white or gray vest and providing some training and demonstrating how to use the tablet and MyChart Bedside. I want to talk with you today about what you thought about that training and about MyChart Bedside.

- Before your most recent hospital stay, had you used MyChart or any outpatient portal before? [Remind the patient that MyChart is the portal that they can use at home and that it is different from MCB, which is the one that they used on the tablet while they were a patient in the hospital.]
    - How comfortable are you with MyChart (or a different outpatient portal)?

- When you were in the hospital, were you (or your patient) the primary user of MyChart Bedside?

Section 2: MANAGING YOUR HEALTH

- How do you generally keep track of your health when you are not in the hospital? (i.e., meds, doctors visits, general health status)

- How does that change when you are in the hospital?

Section 3: EXPERIENCE WITH MYCHART BEDSIDE

- How often did you use the MyChart Bedside application on the tablet that you were given when you were admitted? [If they don't remember the tablet, tell them it looks like an iPad and you may have ordered your meals using it.]

- Tell me about how you used MyChart Bedside.
    - Did you look at your daily schedule?
    - Did you view your test results?
    - Did you look at information about your care team?
    - Did you read any educational materials?
    - Did you request any services?
    - Did you make notes about your care?

- What did you like about using MyChart Bedside?
    - Was it easy to use?

- What did you not like about using MyChart Bedside?
    - Did you encounter any problems?

- How did using MyChart Bedside influence how you felt about taking care of yourself once you were discharged?

- What concerns did you have about using MyChart Bedside?

- What would you change about MyChart Bedside?
    - Are there additional features you think might be useful?

Section 4: COMMUNICATION WITH HOSPITAL STAFF

- Have you ever been admitted to any hospital prior to this admission?
    - If yes, how did you keep track of your test results or communications with your care team without MyChart Bedside?
    - How did having MyChart Bedside during this admission compare to your other experiences?

- Did you know who was on your (his or her) care team?
    - How did you know who was on the team?
    - What happened when members of the team changed?

- If you had a question about your (his or her) care while you were in the hospital, whom did you ask?
    - How did you make your request? (e.g., MyChart Bedside message, call button, call the nurse?)
    - Was the response adequate?
    - Was the response timely?

- In what ways did using MyChart Bedside impact how you communicated with your doctors and nurses?
    - How?
    - In what way(s)?

Section 5: EXPERIENCE WITH MYCHART BEDSIDE TRAINING

- What kind of training did you receive about using MyChart Bedside while you were in the hospital?
    - Did a person talking with you provide the training or did you receive written materials?

- What did you think about the training you received for using the MyChart Bedside patient portal?
    - Were all of your questions answered?
    - Were there topics you wish had been covered?

    - Would you recommend this training to other patients?
    - What could we do to improve this training?

- How do you think this training influenced how you used MyChart Bedside during your hospital stay?

- Do you intend to use MyChart [the outpatient portal that you can use from home or on your phone] now that you (he or she) have been discharged from the hospital?
    - Why or why not?
    - Did using MyChart Bedside influence your opinion about MyChart?

INTERVIEW CLOSURE AND FOLLOW-UP

- Is there anything else you would like to tell us about using MyChart Bedside?

Patient interview guide – 6 months postdischarge

BACKGROUND
First, let us THANK YOU for agreeing to participate in our research project. I am (NAME) and I am a researcher from The Ohio State University. I am contacting you as part of a project we are doing to study MyChart Bedside (MCB), a patient portal offered by The Ohio State University Wexner Medical Center (OSUWMC) and the hospitals associated with the medical center.

As part of this study, we are interested in collecting information from patients about how they tracked their health conditions during their inpatient stay and later after they were discharged. Ultimately, this will help us in the future to understand the issues involved so that we can improve patient training and technology based on user experiences.

When you were recently in the hospital, you agreed to participate in this study, and I am calling you as a follow-up to see if you would be interested in participating in a brief telephone interview that will take about 20 minutes. In this interview, we will ask you questions about your experience with managing your conditions while you were in the hospital, including your perceptions about what might be helpful in the future. As a token of appreciation for your time participating in this interview, we will mail you a $15.00 gift card.

OVERVIEW OF INTERVIEW TOPICS
For this interview, I will ask you a series of open-ended questions to get your perspectives about different topics. As an overview, these topics are:

- Section 1: Experience with and Perceived Benefits of MyChart Bedside
- Section 2: Experience with and Perceived Benefits of MyChart

INTRODUCTION TO THE INTERVIEW
We have scheduled the next 20 minutes to discuss these topics. Before we begin the discussion, we need to take you through an informed consent process. In particular, let me make sure that you understand that:

a. Your participation is completely voluntary. If you do choose to talk with us, you may end the interview at any time.
b. We consider this discussion to be confidential. Your participation is confidential in the sense that your name will not be used in any reports or articles.
c. We would also like to record the interview for the purposes of data collection for our research. The recording will not be used to identify you in any way.
d. Do you have any questions about our study or this interview process? Are you okay being recorded?

I will collect your address at the end of this call to mail you your gift card.

PATIENT (OR CAREGIVER) INTERVIEW QUESTIONS

Section 1: EXPERIENCE WITH AND PERCEIVED BENEFITS OF MYCHART BEDSIDE

MyChart Bedside is an electronic personal health record that patients can access using a tablet. It allows patients to see who is on their care team, view their schedules each day, ask questions, view test and lab results, and make notes. OSUWMC has made MyChart Bedside available to most of their hospitalized patients, and we are interested in learning about patients' experiences with this new technology.

- You first joined our study when you were in the hospital about 6 months ago. Have you been a patient in the hospital again since then?
    - If yes, did you use MyChart Bedside during that admission (those admissions)?
    - If no, when you were in the hospital 6 months ago, did you use MyChart Bedside?

Thinking about any of the times you were in the hospital and used MyChart Bedside, I'd like to know about how you used MyChart Bedside.

- What did you like about using MyChart Bedside?
    - Was it easy to use?

- What did you not like about using MyChart Bedside?
    - Did you encounter any problems?

- Would you recommend MyChart Bedside to other patients?

- Did you receive any training while you (your patient) were in the hospital on how to use MyChart Bedside?
    - If yes, what did you think about the training?
        - Did the training help you to understand how to use MyChart Bedside?
        - What would you change about the training?
        - What would you keep?
    - If no, would training have helped you?

- What benefits did you think you got from using MyChart Bedside in the hospital?
    - How?
    - In what way(s)?

Section 2: EXPERIENCE WITH AND PERCEIVED BENEFITS OF MYCHART

MyChart is an outpatient portal offered by OSUWMC that patients can use outside the hospital to track their health conditions, communicate with their doctors, view test and lab results, and schedule appointments. This is available to any OSUWMC patient who wishes to create an account, or any patient of an OSU physician.

- Do you use MyChart now?
  - If you do use MyChart, how do you use it?
  - For how long have you been using MyChart?
  - How did using MyChart Bedside in the hospital influence using MyChart outside of the hospital?

- Did you receive any training in how to use MyChart?
  - If yes, what did you like about the training?
    - Did the training help you to understand how to use MyChart?
    - What would you change about the training?
    - What would you keep about the training?
  - If no, would training have helped you?

- In what ways do you use MyChart outside of the hospital?

- Do you think there are benefits to using MyChart to manage your (your patient's) health conditions?
  - How?
  - In what way(s)?

- Did you or do you have any concerns about using MyChart?

- How does using MyChart compare with other ways you have used to manage your (your patient's) health conditions?

- Would you recommend using MyChart to other patients?

INTERVIEW CLOSURE AND FOLLOW-UP

- Is there anything else you would like to tell us about using MyChart Bedside or MyChart with respect to your experience?
